# A newly identified small tRNA fragment reveals the regulation of different wool types and oxidative stress in lambs

**DOI:** 10.1038/s41598-023-36895-7

**Published:** 2023-06-23

**Authors:** Jiankui Wang, Guoying Hua, Xue Yang, Letian Zhang, Yuhao Ma, Qing Ma, Rui Li, Keliang Wu, Yaofeng Zhao, Xuemei Deng

**Affiliations:** 1 State Key Laboratory of Animal Biotech Breeding & Key Laboratory of Animal Genetics, Breeding and Reproduction, Ministry of Agriculture & Beijing Key Laboratory for Animal Genetic Improvement, China Agricultural University, Beijing, 100193 China; 2 Animal Science Institute of Ningxia Agriculture and Forestry Academy, Yinchuan, 750002 China; 3 Jinfeng Animal Husbandry Group Co., Ltd., Chifeng, 024000 China

**Keywords:** Agricultural genetics, Animal breeding, Development, Epigenetics

## Abstract

Novel small RNAs derived from tRNAs are continuously identified, however, their biological functions are rarely reported. Here, we accidentally found the reads peak at 32nt during statistical analysis on the miRNA-seq data of lamb skin tissue, and found that it was related to the wool type of lambs. This 32nt peak was composed of small tRNA fragments. The main component sequence of this peak was a novel small tRNA derived from Glycyl tRNA (tRNA^Gly^), the expression level of tRNA^Gly^-derived tRNA fragments (tRF^Gly^) was 5.77 folds higher in the coarse wool lambs than that in the fine wool lambs. However, in contrast, the expression of tRNA^Gly^ in the skin of fine wool lambs is 6.28 folds more than that in coarse wool lambs. tRNA^Gly^ promoted the synthesis of high glycine protein including KAP6 in fine wool lamb skin. These proteins were reported as the major genes for fine curly wool. Integrative analysis of target gene prediction, proteomics and metabolomics results revealed that tRF^Gly^ reduced the level of reactive oxygen species (ROS) in the skin of coarse wool lambs by targeted inhibition of the Metabolic signal and the corresponding Glutathione metabolic pathway, on the contrary, the level of oxidative stress in the skin of fine wool lambs was significantly higher. This study revealed for the first time the relationship between tRNA^Gly^ and its derived tRF^Gly^ and animal traits. tRF^Gly^ has the function of targeting and regulating protein synthesis. At the same time, tRF^Gly^ can reduce the expression of its resource complete tRNA, thereby reducing its ability to transport specific amino acid and affecting the expression of corresponding proteins.

## Introduction

Transfer RNA (tRNA) is a type of non-coding RNA^1^. Most tRNAs consist of short, clover-shaped chains of seventy to ninety nucleotides that are folded to carry amino acids into the ribosome for protein synthesis under the guidance of mRNA^2^. tRNA can recognize the codon on the mRNA by its own anticodon and translocate the amino acid corresponding to that codon to the polypeptide chain in ribosome synthesis^3^. Each tRNA molecule can only attach to one amino acid theoretically, the lack of tRNA molecules usually affects the efficiency of translation.

In addition to its key role in protein synthesis, tRNA is gradually being recognized for its function as a potential molecular precursor for gene expression regulation^4^. Recent findings suggest that some small fragments of tRNA-derived RNAs have been identified by high-throughput sequencing in a variety of cell lines. These sheared products are thought to have the ability to interact with a variety of key molecules in the microRNA processing system such as the Dicer, Ago family of proteins^4^. It has been found that these small molecule RNAs derived from tRNAs can be broadly classified into two categories: the first category requires Dicer to participate in its formation as long as it originates from the 3′ terminal part of mature tRNAs. The second type is derived from the 3′ terminal part of the tRNA precursor and does not require Dicer to participate in its formation, but requires RNaseZ, which is required for the normal processing of another type of tRNA precursor into mature tRNA^5^. Meanwhile, there is also some evidence that the 5′ end sequences of certain tRNAs can also be processed into such tRNA fragments (tRFs), such as miR-1308 and miR-886-5p corresponding to tRF^Gly^ and tRF^Ala^ in the miRNBase database, however, there are relatively few studies on these tRFs derived from the 5′ end sequences of tRNAs^6^.

The study on the function of tRFs on animal phenotype is rare, this may be because it is more suitable for traits with epigenetic characteristics. In our recent research, we noticed a special phenomenon that not the newborn lambs of fine wool sheep are covered with fine and curly wool. A small number of newborn lambs are coarse wool lambs covered with long and straight coarse wool. But when they grow up, they will become undistinguishable fine wool sheep. When we studied their miRNA differences in skin tissues, we unexpectedly found that there was a significant difference in the reads count of small tRNA fragments between coarse wool lambs and fine wool lambs. Therefore, we tracked this difference and further found that the core difference of tRF was concentrated in tRF^Gly^ (tRNA Fragment derived from tRNA^Gly^). Based on proteome and metabolome analysis using the same skin tissues of miRNA-seq, we identified tRF^Gly^ target molecules and metabolites, and found that the unbalanced expression of tRNA^Gly^ and tRF^Gly^ in skin tissue regulates wool development and skin tissue adaptability. This study provides a way of small tRNA analysis and an example of small tRNA function.

## Results

### Coarse-wool phenotypic lambs emerge during fine wool sheep breeding

**Figure 1:**
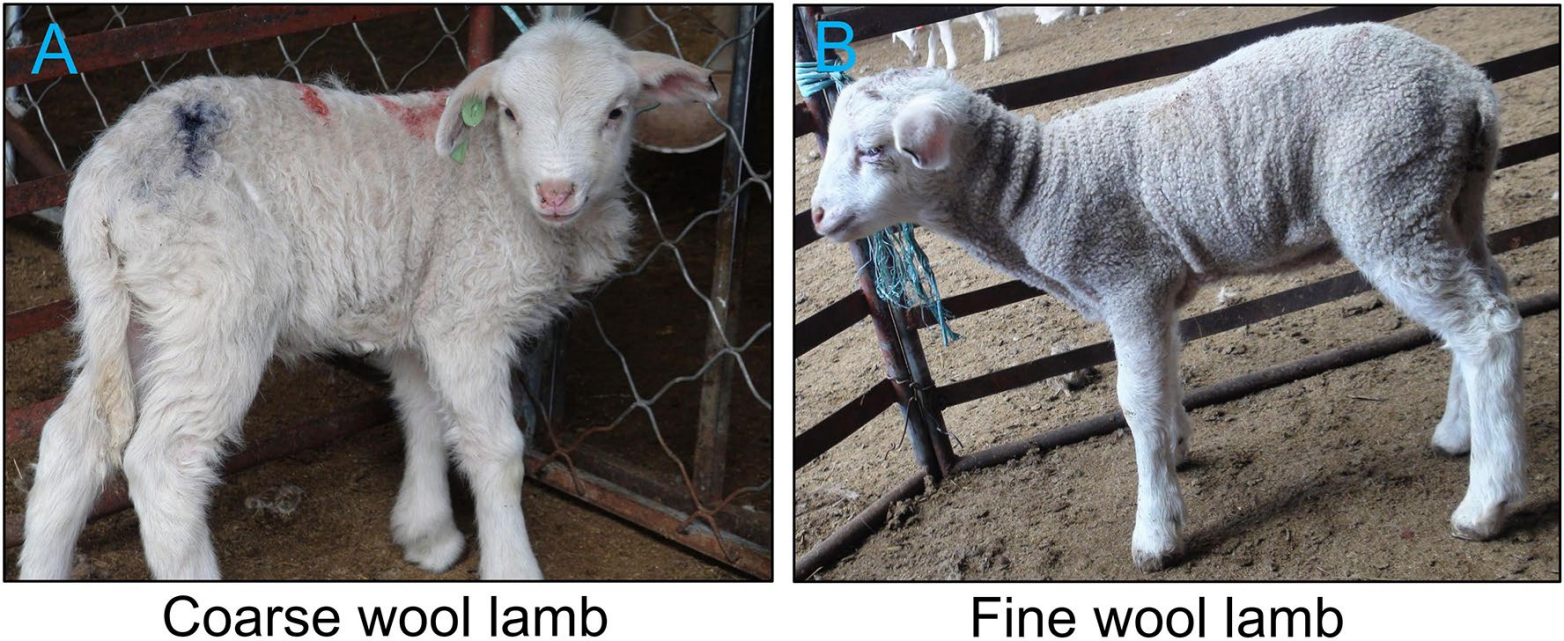
Sibling lambs with different wool types. (**A**) The coarse wool lamb, (**B**) the fine wool lamb from the same fine wool sheep population.

In a super-ovulation breeding population of fine wool sheep, coarse wool lambs appear repeatedly in multiple generations. In the 4-year statistics, we found that the probability of coarse wool lambs is about 5%. The coarse wool newborn lambs have long and dense wool (Fig. 1A), while that of the fine wool lambs is short and curly (Fig. 1B). The coat of coarse wool lambs has higher coverage on the skin, so it is conducive to keeping the lambs warm, more resistant to low temperature, and their body size is larger and stronger compare to fine wool lambs. However, both coarse wool lambs and fine wool lambs all show typical characteristics fine wool sheep after they grow up. There is no significant difference in wool diameter and curl at adulthood, while coarse wool lambs have better environmental adaptability in infancy.

### An anomalous reads peak at 32nt discovered in the miRNA-seq data of coarse wool group

**Figure 2:**
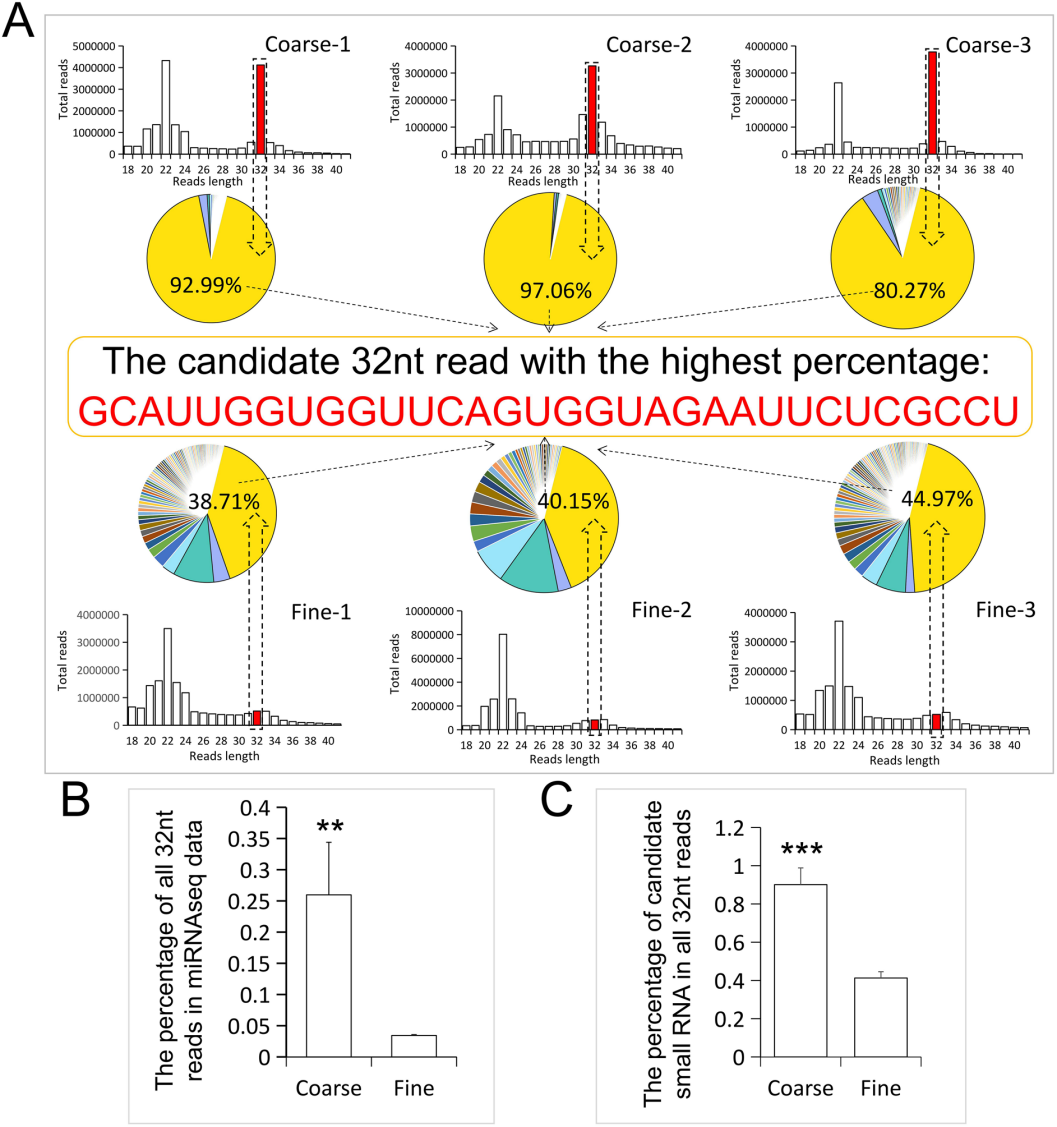
A newly identified 32nt small RNA in miRNA-seq data. (**A**) A novel 32nt small RNA was identified by analyzing the length distribution of miRNA-seq data reads. The column charts show the length distribution of miRNA-seq reads in coarse and fine wool lambs skin tissues, in which the X-axis represents reads length, while the Y-axis represents reads counts. The red bars indicate the 32nt peak of the miRNA-seq data. The pie charts show the percentage of different sequences at 32nt. The sequence displayed in the middle section is the small RNA sequence with the highest percentage at 32nt. (**B**) Comparative analysis of the number of all reads at 32nt between coarse and fine wool lambs. (**C**) Comparative analysis of the difference in the count of candidate reads with the highest proportion at 32nt between coarse and fine wool tissues.

We analyzed clean reads with lengths of 18–41nt when comparing miRNA-seq data of skin tissues of coarse wool and fine wool lambs. Unexpectedly, we found a 32nt sub-peak in coarse wool lambs which was almost at the same level as the classical microRNA reads of 22nt miRNAs (Fig. 2A). Interestingly, in fine wool lambs, the reads peak at the same position is significantly lower than that in coarse wool lambs (Fig. 2A and B). We speculate that this subset of small RNA molecules is related to the coarse wool and fine wool phenotype and has correlation with related characteristics of lambs. The 32nt small RNA was collected and the sequences were further analyzed. The results showed that the reads at this position contain mainly four different 32nt small RNA sequences, with a small amount of base polymorphisms (Table S1). Most importantly, the results also showed that there was a base-consistent sequence in all 32nt small RNAs (Fig. 2A), and the reads count of this sequence ranked first among all sequences. In the coarse wool group, it accounted for more than 90% of all 32nt reads, which was significantly higher than the percentage of this sequence in the fine wool group (Fig. 2A and C).

### The newly identified 32nt small RNA is derived from glycine tRNA

**Figure 3:**
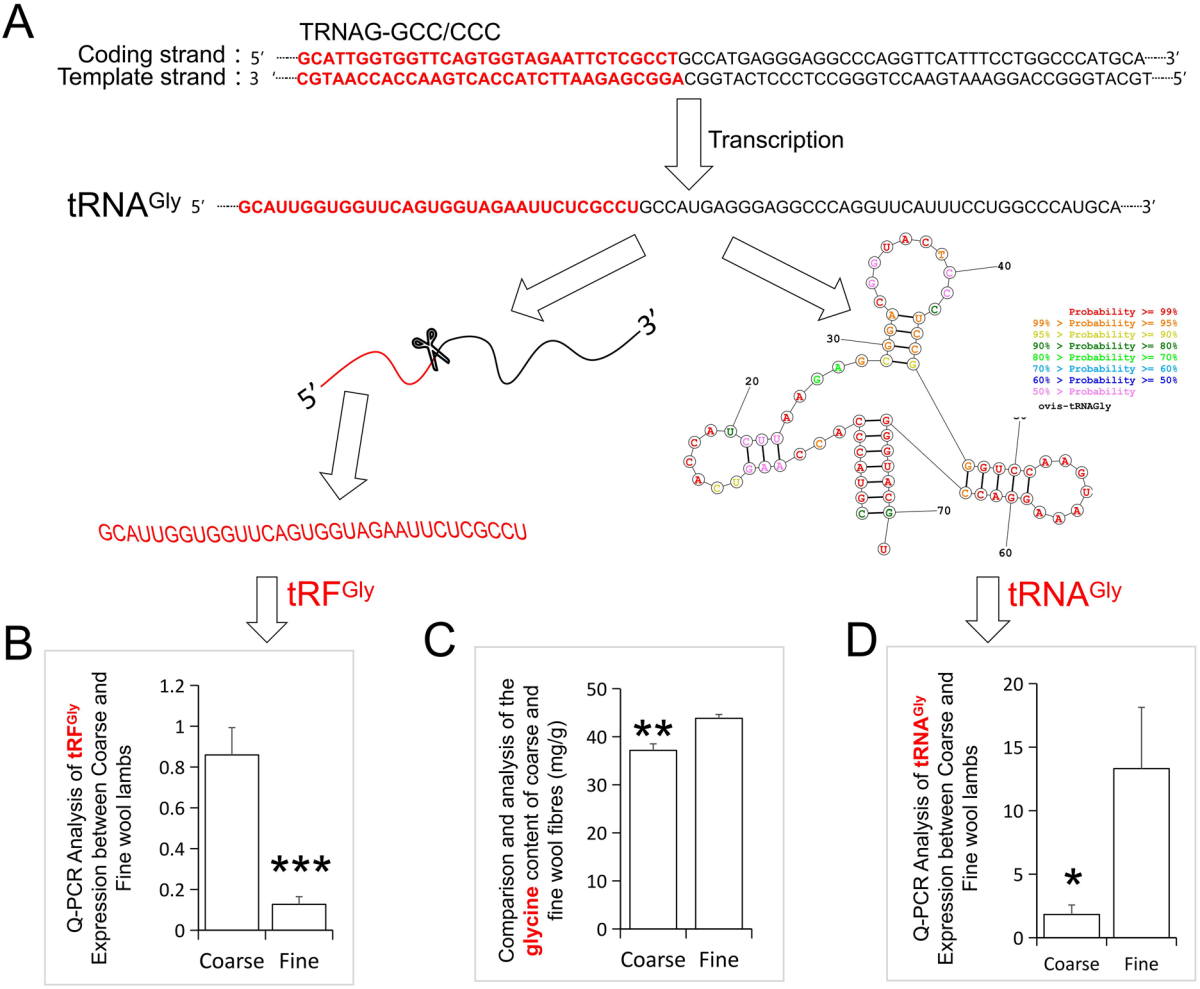
Combined analysis of tRF^Gly^ and tRNA^Gly^ expression differences in the coarse and fine wool groups. (**A**) The formation process of tRF^Gly^ and tRNA^Gly^, the prediction of RNA secondary structure was performed by online tool ProbKnot (http://rna.urmc.rochester.edu/RNAstructureWeb/Servers/ProbKnot/Example.php). (**B**) The relative expression level of tRF^Gly^ between coarse and fine wool lambs, ***, P < 0.0001. (**C**) The relative glycine level of wool fibres between coarse and fine wool lambs **, P < 0.01. (**D**) The relative expression level of tRNA^Gly^ between coarse and fine wool lambs, *, P < 0.05.

To determine the origin of the novel 32nt small RNA, we aligned the 32nt sequence to sheep reference genome (GCF_002742125.1_Oar_ rambouillet_v1.0), and found that the sequence was aligned to 15 positions in the sheep genome (Table 1). All of these 15 loci are located at the 5′ end of the TRNAG-CCC/TRNAG-GCC (Fig. 3A), which is the tRNA gene corresponding to the glycine codon CCC and GCC. Therefore, our newly identified 32nt small RNA is a fragment derived from Glycyl tRNA (tRNA^Gly^), here, the 32nt small Glycyl tRNA fragment was abbreviated as tRF^Gly^ (Fig. 3A). Q-PCR analysis verified that the expression level of tRF^Gly^ in the coarse wool lambs was extremely significantly higher (5.77 folds) than that in fine wool lambs (Fig. 3B). However, when detecting the expression level of the corresponding complete tRNAGly, it was found that the expression level in coarse wool group was significantly lower (6.28 folds) than that in the fine wool group (Fig. 3D). In addition, Amino acid levels were also measured using wool fibres of coarse and fine wool lambs and the results showed that the fine wool lambs showed higher levels of glycine than that of the coarse wool lambs, with highly significant differences (Fig. 3C).

**Table 1:** Sequence alignment of the highest proportion of 32nt reads to the sheep reference genome.

Chr	Start	End	Strand	Length	Gene_name
NC_040252.1 (Chr 1)	105273828	105273898	−	71	TRNAG-CCC
NC_040252.1 (Chr 1)	105275606	105275676	+	71	TRNAG-CCC
NC_040252.1 (Chr 1)	105276143	105276213	−	71	TRNAG-CCC
NC_040252.1 (Chr 1)	105289175	105289245	+	71	TRNAG-CCC
NC_040252.1 (Chr 1)	105289720	105289790	−	71	TRNAG-CCC
NC_040252.1 (Chr 1)	105291492	105291562	+	71	TRNAG-CCC
NC_040252.1 (Chr 1)	105392585	105392655	−	71	TRNAG-CCC
NC_040252.1 (Chr 1)	105478889	105478959	−	71	TRNAG-CCC
NC_040252.1 (Chr 1)	105505558	105505628	+	71	TRNAG-CCC
NC_040252.1 (Chr 1)	105587574	105587644	−	71	TRNAG-CCC
NC_040252.1 (Chr 1)	120100333	120100403	−	71	TRNAG-GCC
NC_040253.1 (Chr 2)	165210771	165210841	+	71	TRNAG-GCC
NC_040259.1 (Chr 8)	7062119	7062189	−	71	TRNAG-CCC
NC_040262.1 (Chr 11)	35658466	35658536	−	71	TRNAG-GCC
NC_040265.1 (Chr 14)	946283	946353	−	71	TRNAG-GCC
NC_040265.1 (Chr 14)	946941	947011	−	71	TRNAG-GCC
NC_040265.1 (Chr 14)	953972	954042	+	71	TRNAG-GCC
NC_040265.1 (Chr 14)	1344225	1344295	+	71	TRNAG-GCC
NC_040271.1 (Chr 20)	33381996	33382066	+	71	TRNAG-GCC
NC_040271.1 (Chr 20)	34261573	34261643	+	71	TRNAG-GCC

### tRF^Gly^ targets and regulates glutathione-related metabolic pathways

**Figure 4:**
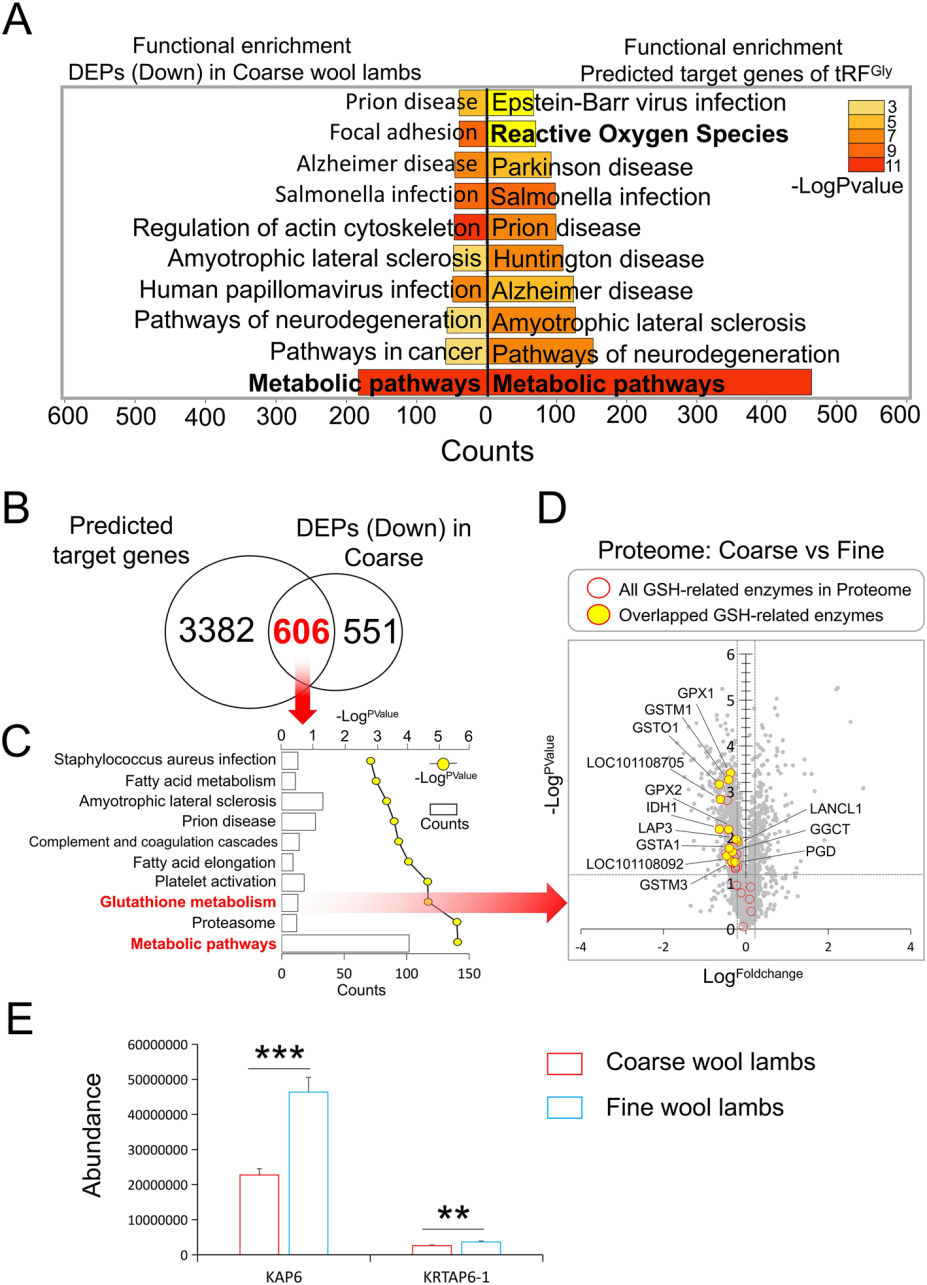
Integration analysis of proteomic data and tRF^Gly^ target gene prediction results. (**A**) Comparative functional enrichment analysis between tRF^Gly^ predicted target genes and low-expressed proteins in coarse wool group of the proteome (https://david.ncifcrf.gov/tools.jsp). (**B**) The overlapped genes between tRF^Gly^ predicts target genes and low-expressed proteins in coarse wool group of the proteome. (**C**) Functional enrichment analysis of the overlapped genes in (**B**) (https://david.ncifcrf.gov/tools.jsp). (**D**) Volcanol plot of differentially expressed proteins in proteomic. (**E**) The relative expression of candidate proteins based on the proteome data, ***: P < 0.0001, **: P < 0.001. (**E**) Comparison of the expression levels of HGTP (KAP6 and KRTAP6-1) in the skin of Coarse and Fine wool lambs.

Studies have shown that tRFs has the ability to post-transcriptional regulation^7–9^. So we used Miranda software to predict the target genes of tRF^Gly^, and the results showed that a total of 3988 genes were potential target genes of tRF^Gly^ (Supplementary Table S2). Functional enrichment analysis of these genes using David’s online tool showed that 435 genes were enriched to metabolic signaling pathway, which ranked first among all enriched signaling pathways (Fig. 4A). In addition, the reactive oxygen species (ROS) pathway, consisting of 70 genes, was enriched in the top 10 positions (Fig. 4A).

TMT-tagged proteome detected a total of 4757 differentially expressed proteins between the coarse and fine wool groups (Table S3). Among them, 372 differentially expressed proteins were significantly up-regulated and 1157 differentially expressed proteins were significantly down-regulated in coarse wool group (Table S4). In proteome data, functional enrichment analysis using David's online tool revealed many results similar to the signaling pathways enriched by predicted tRF^Gly^ target genes. Especially, 183 down-regulated proteins in the coarse wool proteome were enriched in the metabolic signaling pathway, which also ranked first, and was consistent with the result of tRF^Gly^ target genes predicted signaling pathway (Fig. 4A). In view of the miRNAs, including tRFs, mainly act as post-transcriptional inhibitors of target genes^7^. We speculate that the corresponding down-regulated differentially expressed proteins (DEPs) in coarse wool group reflects, to some extent, the result of post-transcriptional translational repression of tRF^Gly^ target genes. In fact, a total of 606 genes co-occurred among the predicted target genes of tRF^Gly^ and the down-regulated DEPs in coarse wool group, and these overlapping genes accounted for 52.38% of all the down regulated DEPs in the coarse wool lambs (Fig. 4B). In addition, functional enrichment analysis of these 606 overlapping proteins revealed the top ten signaling pathways dominated by metabolic signals (Fig. 4C), notably the glutathione metabolic pathway corresponding to the reactive oxygen pathway was also enriched to the third place, which includes numerous glutathione transferases (GST), glutathione reductases (GSH) and glutathione peroxidases (GPx) (Fig. 4A, C and D).

We noticed that the 32nt tRF^Gly^ reads count of the fine wool lamb group was far lower than that of the coarse wool lamb group, while the expression of tRNA^Gly^ of the fine wool group was significantly higher than that of the coarse wool group. In the proteome data, we found that the high glycine tyrosine protein (HGTP) was highly expressed in the skin tissues of fine wool lambs (Fig. 4E), including but not limited to KAP6 and KRTAP6-1 genes, which were reported as the major genes of fine wool in sheep^10–12^(Table S2).

### Metabolomic analysis reveals high levels of oxidative stress in skin tissues of fine-wool lambs

**Figure 5:**
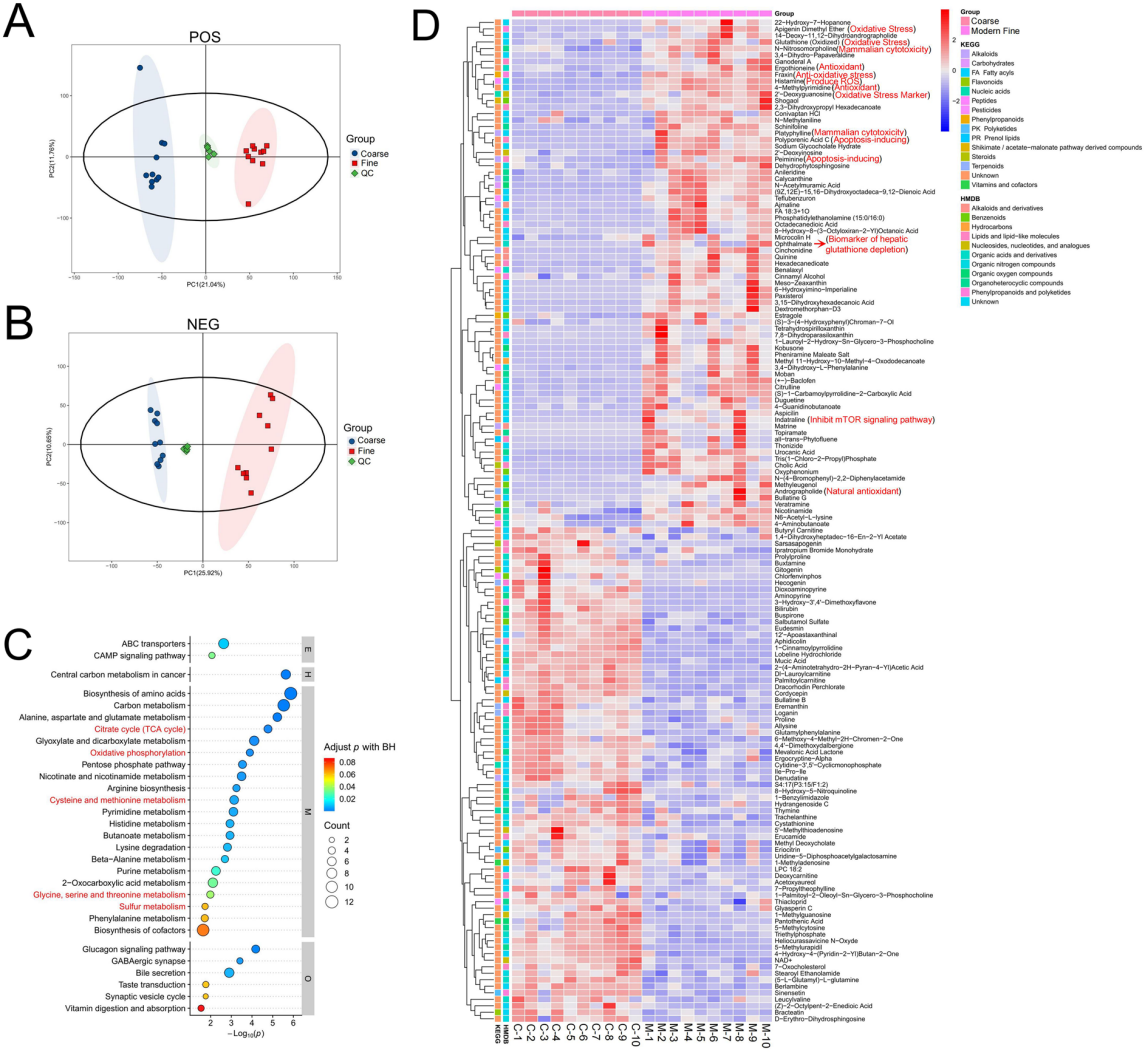
Metabolomic analysis of coarse and fine wool lamb groups. (**A**) Principal Component Analysis (PCA) of Metabolome data according to positive ion metabolites. (**B**) PCA of Metabolome data according to negative ion metabolites. (**C**) Functional enrichment analysis of low-level metabolites in the coarse wool group. (**D**) The heatmap of top differential metabolites between coarse and fine wool lambs, C-1 to C-10 indicate the Coarse wool lambs, M-1 to M-10 indicate the modern Fine wool lambs.

Proteome results firstly enriched the metabolic signaling pathway. Therefore, we performed a metabolome comparison between the coarse and fine lamb skin tissues. The results of the principal component analysis (PCA) showed that the coarse and fine wool lambs were completely separated, while the Quality Control (QC) samples were clustered together, indicating the high quality of the metabolomic data (Fig. 5A and B). Functional enrichment analysis of down-regulated metabolites showed a lower metabolic level in the skin of coarse wool lambs than that of fine wool lambs, which was consistent with the proteomic results (Figs. 5C and 4A). In addition, the glycine metabolic pathway was enriched by the down-regulated metabolites of coarse wool group. On the contrary, the fine wool group had a stronger glycine metabolism (Fig. 5C), which again supported the higher expression level of tRNA^Gly^ gene (Fig. 3C) and the higher glycine protein expression in fine wool lambs in the aforementioned results. In addition, cysteine, methionine and sulfur metabolism were enriched in the down-regulated metabolites of the coarse wool group (Fig. 5C), all of which were closely associated with oxidative stress^13–15^. More importantly, the results of differential metabolite analysis revealed more metabolic molecules related to oxidative stress and reactive oxygen species metabolism in the fine wool group. These molecules were at the top of all metabolites in terms of both fold-change and P-value between the coarse and fine wool groups (Fig. 5D and Tables S5 and S6), for instance, Oxidized Glutathione^16–19^, Apigenin Dimethyl Ether^20^, *N*-Nitrosomorpholine^21,22^, Ergothioneine^23–26^, Fraxin^27,28^, Histamine^29–31^, 2′-Deoxyguanosine^32,33^, Ophthalmate^34^, and Andrographolide^35^. This shows that there are more oxidative stress reactions in the skin of fine wool lambs.

## Discussion

In this study, we identified a novel 32nt small RNA peak, which contains a tRF mainly derived from glycine tRNA. We found that the expression level of tRF^Gly^ in the coarse wool group was significantly higher than that in the fine wool group, while the expression level of tRNA^Gly^ was significantly higher in the fine wool group than in the coarse wool group. Therefore, we speculate that there are two metabolic directions of glycine tRNA in lamb skin tissues, one is to transport glycine to help synthesize the corresponding proteins, and this metabolic direction mainly occurs in fine wool lamb skin tissues. The other metabolic direction is targeted regulation of small tRF molecule, this process mainly occurs in the coarse wool lamb skin tissues.

Among the corresponding proteins synthesized by tRNA^Gly^ transporting glycine, high glycine-tyrosine protein (HGTP) is a well-known wool trait associated keratin^36^. The expression of HGTP family member genes KAP6, KAP7 and KAP8 has an important influence on the fineness and curvature of wool^37^. HGTP and high sulfur proteins (HSP) are expressed in the positive and paracortical regions of wool fibers, respectively, and this symmetrical distribution of positive and negative cortices create the curvature of fine wool^38–40^. In fact, many studies related to genome-wide association study have located KRTAP6-1, suggesting that this gene is closely associated with fine wool traits^10–12,41^. In the proteomic results of this study, we found that one of the HGTP proteins, KRTAP6-1, was highly significantly overexpressed in the fine wool group, most likely due to the high expression level of tRNA^Gly^ in the skin of fine wool lambs which enhanced glycine transport and thus increased the synthesis of HGTP-related proteins. Metabolomic analysis also showed that the down-regulated metabolites of the coarse wool group were enriched in glycine metabolic pathway. In fact, when measuring the glycine levels in the wool fibres of coarse and fine wool lambs, the fine wool group did contain higher levels of glycine. This further suggests that the reduction of intact tRNA^Gly^ in the skin tissues of the coarse wool group is likely to reduce its glycine transport capacity compared with the fine wool group, which in turn further explained the higher expression of HGTP (i.e. KRTAP6-1) in the fine wool group. This may be one of the biochemical reasons for the difference in wool type between the coarse wool and fine wool lambs in this study.

In another small peptide synthesized by tRNA^Gly^ transporting glycine, glutathione is a tripeptide containing γ-amide bond and a sulfhydryl group, composed of glutamic acid, cysteine and glycine, and is present in almost every cell of the body^42^. Glutathione helps to maintain normal immune system function, and has antioxidant and integrative detoxification effects^43^. Glutathione is available in reduced (G-SH) or oxidized (G-S-S-G) form. Reduced glutathione predominates under physiological conditions^43–46^. Glutathione reductase can catalyze the interchange between the two types^47^. The proteome of this study showed high expression of glutathione transferase and reductase in the fine wool group. The metabolomic results also showed that the level of oxidized glutathione,a markers of oxidative stress^48^, in the fine wool group was extremely significantly higher than that in the coarse wool group, indicating that a stronger oxidative stress reaction was generated in the skin tissues of the fine wool lambs.

The lambs in this study are all from the same fine wool sheep breeding population. Coarse wool lambs occasionally appear in newly born sheep. It is believed that the wool diameter of such lambs is thick after adulthood. Therefore, coarse wool lambs are usually eliminated and only fine wool lambs are retained. Most of the fine wool sheep production areas in China are located in the north, and the lambing periods are mostly concentrated in cold winter and early spring^49^. The poor adaptability of fine wool lambs affects the early survival and growth of lambs, which should be related to the short and sparse covering of fine wool lambs, which is not conductive to cold resistance. In this study, it was found that there were high levels of oxidative stress metabolites such as oxidative glutathione in the skin tissue of fine wool lambs, which should be a defense mechanism developed by fine wool lambs to overcome harsh environment including low temperature and wet weather by enhancing oxidative stress response.

The coarse wool lambs and fine wool lambs analyzed in this study come from a half-sibling family. It is observed that there is no significant difference in wool diameter between them after adulthood (data not shown). Coarse wool lamb did not produce strong oxidative stress reaction. The high expression of tRF^Gly^ has a down-regulation effect on the metabolic pathway and oxidative stress response of coarse wool lamb skin tissue. This may also represent the natural adaptability of coarse wool lambs to cold environment. The trade-offs between tRNA and tRF in coarse wool lambs and fine wool lambs exactly correspond to the differences in wool type and adaptation to the environment at early stage.

In this study, a novel tRF^Gly^ was identified as the reason for the wool type differences and an important regulatory molecule for environmental adaptation in newborn lambs. The functional mechanism of tRFs need further study.

## Methods

### Animals and samples

Three coarse wool lambs and three fine wool lambs originated from the same fine-wool sheep population in Inner Mongolia, China. They are of the same sex and come from the same half-sibling family obtained by supernumerary ovulation and embryo transfer technique. The age of the six experimental lambs was 30 days old. Their skin tissues were rapidly placed in liquid nitrogen and their total RNA and proteins were extracted for miRNAseq, Q-PCR, and proteome, respectively. Metabolome was performed with 10 coarse wool type lambs and 10 fine wool type lambs from the same fine wool sheep population, which were obtained in four consecutive years of embryo transfer experiments. They were sex-discordant half-sibling lambs.

All procedures on sheep performed in this study were approved by the Animal Care and Use Committee of China Agricultural University (Approval no. XK257), all methods were reported in accordance with ARRIVE guidelines (https://arriveguidelines.org), and all methods were carried out in accordance with relevant guidelines and regulations.

### Determination of amino acid levels in wool

Wool fibres were collected from six separate lambs (three coarse wool lambs and three fine wool lambs), 10 mg of wool fibres was taken and the wool fibres were shredded with scissors. Carefully added 4 mL of 1:1 analytically pure hydrochloric acid (6 mol/L). The tube was sealed by blowing nitrogen on a nitrogen blower for 15 min. The sealed tubes were hydrolysed in an oven at 110 ℃ for 30 h and then cooled and opened. The sample was fixed to 100 mL. Aspirate 2 mL of the volume of the sample accurately and deacidify on a nitrogen blower, temperature 60 °C and leave to dry. The amino acids were accurately mixed on a 0.02 mol/L HCL vortexer, passed through a 0.22 µm filter column and the amino acids were determined for each sample in an amino acid analyser (Hitachi, L-8900, Japan).

### MiRNA-seq and novel tRF^Gly^ identification

Total RNA was extracted using TRIZOL and purity was examined by NanoDrop 2000. RNA integrity was assessed by 1% agarose gel electrophoresis. Then, a small RNA library was constructed with 1 μg total RNA for each sample using TruSeq Small RNA Sample Prep Kits (Illumina, San Diego, CA, USA). The sequencing was subjected to Illumina Hiseq2000. Raw reads were provided in the FASTQ format and were filtered out by removing adaptors. We retained clean reads whose length was > 18nt. We performed reads length distribution statistics using fastqc software (https://www.bioinformatics.babraham.ac.uk/projects/fastqc/), and the interval of length was mainly 18–40nt. Interestingly, compared coarse wool group and fine wool group, we find that significant differentially read length was 32nt. Therefore, we extracted the sequence of the 32nt using SeqKit software (https://bioinf.shenwei.me/seqkit/). The 32nt reads were mapped to the sheep genome (GCF_002742125.1_Oar_rambouillet_v1.0) using bowtie^50^ and quantitation by featureCounts^51^. The differential expression of 32nt reads was based on the expression counts by t-test method. We mapped the differential expression of 32nt reads to the sheep genome (Oar_rambouillet_v1.0) by BLAST software^52^. The annotation of 32nt reads was performed by NCBI database (https://www.ncbi.nlm.nih.gov/). Miranda^53^ was used to find genomic targets for 32nt reads.

### Proteome

Skin tissues were suspended on ice in 200 μL lysis buffer (4% SDS, 100 mM DTT, 150 mM Tris–HCl pH 8.0), then disrupted with agitation using a homogenizer, and boiling for 5 min. The tissues were further ultrasonicated and boiling again for another 5 min. After centrifugated at 16,000 rpm for 15 min, the supernatants were collected and quantified with a BCA Protein Assay Kit (Bio-Rad, USA). Digestion of protein (200 μg for each sample) was performed according to the FASP procedure. The peptide concentration was determined with OD280 by Nanodrop device.

Peptides were labeled with TMT reagents according to the manufacturer’s instructions (Thermo Fisher Scientific), and each aliquot was reacted with one tube of TMT reagent, respectively. After incubation, the Multiplex labeled samples were pooled together and lyophilized. TMT-labeled peptides mixture was fractionated using a Waters XBridge BEH130 column (C18, 3.5 μm, 2.1 × 150 mm) on an Agilent 1290 HPLC operating at 0.3 mL/min. Buffer A consisted of 10 mM ammonium formate and buffer B consisted of 10 mM ammonium formate with 90% acetonitrile; both buffers were adjusted to pH 10 with ammonium hydroxide. A total of 30 fractions were collected for each peptide mixture, and then concatenated to 15 (pooling equal interval RPLC fractions). The fractions were dried for nano LC–MS/MS analysis. LC–MS analysis was performed on a Q Exactive mass spectrometer that was coupled to Easy nLC (Thermo Fisher Scientific). Peptide from each fraction was loaded onto a the C18-reversed phase column (12 cm long, 75 μm ID, 3 μm) in buffer A (2% acetonitrile and 0.1% Formic acid) and separated with a linear gradient of buffer B (90% acetonitrile and 0.1% Formic acid) at a flow rate of 300 nL/min over 60 min. Full MS resolutions were set to 120,000 at m/z 200 and the full MS AGC target was 300% with an injection time of 25 ms. Mass range was set to 350–1400. The instrument was run with peptide recognition mode enabled. All raw files were processed in Proteome Discoverer 2.4 (Thermo Fisher Scientific) and were imported into MaxQuant software (version 1.6.0.16) for data interpretation and raw data were searched against the database Uniprot_ OvisAries _23084_ UP000002356. The search followed an enzymatic cleavage rule of Trypsin/P and allowed maximal two missed cleavage sites and a mass tolerance of 20 ppm for fragment ions. The modification set was as following: fixed modification: Carbamidomethyl (C), TMT6plex(K), TMT6plex(N-term), Variable modification: Oxidation(M) and Acetyl (Protein N-term). The minimum 6 amino acids for peptide, ≥ 1 unique peptide was required per protein. For peptide and protein identification, false discovery rate (FDR) was set to 1%. TMT reporter ion intensity were used for quantification.

Analyses of bioinformatics data were carried out with Perseus software, Microsoft Excel and R statistical computing software. Differentially significant expressed proteins were screened with the cutoff of a ratio fold-change of > 1.20 or < 0.83 and P-values < 0.05. Expression data were grouped together by hierarchical clustering according to the protein level. To annotate the sequences, information was extracted from UniProtKB. GO and functional enrichment analyses were carried out using online tool David^54,55^ (https://david.ncifcrf.gov/) with the Fisher’s exact test, and FDR correction for multiple testing was also performed. Enriched GO and signal pathways were nominally statistically significant at the P < 0.05 level.

### Metabolome

In this experiment, the full spectrum of the sample was analyzed by HILIC UHPLC-Q-EXACTIVE MS technology combined with data-dependent acquisition method. At the same time, the primary and secondary mass spectrometry data were obtained. Then Compound Discoverer 3.0 (Thermo Fisher Scientific) was used to perform peak extraction and metabolite identification of the data. To monitor the stability and repeatability of instrument analysis, quality control (QC) samples were prepared by mixing aliquots of the all samples to be a pooled sample. The ACQUITY UPLC BEH C18 column (100 mm*2.1 mm, 1.7 μm, Waters, USA) was used for chromatographic separation. The mobile phase A was water and 0.1% formic acid, B mobile phase is acetonitrile. The loading volume for each sample is 5 μL. The sample was placed in the 4 °C autosampler during the entire analysis. In order to avoid the influence caused by the fluctuation of the detection signal of the instrument, a random order is adopted for continuous analysis of samples. QC samples are injected after each group of samples in the sample queue to monitor and evaluated the stability of the system and the reliability of experimental data.

Detection was used by Electrospray ionization (ESI) positive ion and negative ion modes. The samples were separated by UHPLC and analyzed by Q-Exactive quadrupole-electrostatic field orbitrap high-resolution mass spectrometer (Thermo Fisher Scientific). In the extracted ion features, only the variables having more than 50% of the nonzero measurement values in at least one group were kept. Compound identification of metabolites was performed by comparing of accuracy m/z value (< 25 ppm), and MS/MS spectra with an in-house database established with available authentic standards. After normalized to total peak intensity, the processed data were uploaded, then imported into SIMCA-P (version 14.1, Umetrics, Umea, Sweden), where they were subjected to multivariate data analysis, including Pareto-scaled principal component analysis (PCA) and orthogonal partial least-squares discriminant analysis (OPLS-DA). The robustness of the model was evaluated by the seven-fold cross-validation and response permutation testing. The variable importance in the projection (VIP) value of each variable in the OPLS-DA model was calculated to indicate its contribution to the classification. Metabolites with the VIP value > 1 was further applied to Student’s t-test at univariate level to measure the significance of each metabolite, the P values less than 0.05 were considered as statistically significant. The significant difference metabolites were screened, and then cluster analysis and metabolic pathway analysis were performed on the difference metabolites.

### Q-PCR

Q-PCR experiments for tRNA^Gly^, specifically, cDNA synthesis was performed with 500 ng of total RNA, following the protocol accompanying the PrimeScript™ RT-PCR Kit (Takara, Dalian, China) with the CFX96TM Real-Time System (BIO-RAD, California, USA), particularly necessary to point out that the reverse transcription primers should chose “Random 6 mers” for reverse transcription of total tRNA. The entire Q-PCR was performed in a 20 μL system, containing 1 μL total cDNA, 0.5 μL forward primer: TACCCACCAAGTCACCATC, 0.5 μL reverse primer: CGTACCCGGTCCTTTACTTG, 10 μL qPCR PreMix (TIANGEN, FP209-01, China), 8 μL ddH2O. The reaction was performed for 5 min at 95 °C, followed by 30 s at 95 °C, with a subsequent 40 cycles of amplification (95 °C for 5 s, 60 °C for 10 s and 72 °C for 15 s). Besides, the β-actin was selected for housekeeping gene and the forward primer: GGGACCTGACAGACTACCTCAT, and the reverse primer: TTCTCCTTGATGTCACGCA.

Q-PCR experiments for tRF^Gly^, specifically, the reverse transcription of tRF^Gly^ and U6 was carried out according to the experimental procedure of kit TaqManTM MicroRNA Revverse Transcription Kit (applied biosystems by Thermo Fisher Scientific, USA). Among them, every 15 μL reverse transcription reaction system needs 10 ng total RNA, and the reverse transcription reaction system includes 7 μL Mastermix mixture, 3 μL 5 × RTprimer and 5 μL RNA samples. Q-PCR selected SYBR Green qPCR mix kit (TIANGEN, FP209-01, China) and carried out in CFX96TM Real-Time System (BIO-RAD, California, USA). Reaction procedure: 5 min at 95 °C, followed by 30 s at 95 °C, with a subsequent 40 cycles of amplification (95 °C for 5 s, 58 °C for 10 s and 72 °C for 15 s). The RT primers and U6 primers were purchased from RiboBio biological company (RiboBio, Guangzhou, China), The primers of tRF^Gly^ was also designed and purchased from RiboBio biological company (RiboBio, Guangzhou, China). The repeats of three holes were set for each sample, and U6 was used as the internal reference. The relative expression between groups was calculated by 2^−△△CT^^56^.

### Statistical analysis

Differences between Coarse and Fine wool lamb groups were tested using independent Student’s t-test, two-tailed t-test was conducted with the following P-values: *, P < 0.05; **, P < 0.01; ***, P < 0.001.

## Supplementary Information

Supplementary Table S1.

Supplementary Table S2.

Supplementary Table S3.

Supplementary Table S4.

Supplementary Table S5.

Supplementary Table S6.

## Data Availability

The datasets generated during the current study are available in the NCBI with accession number PRJNA760789 (Coarse-1: SRX12021132, Coarse-2: SRX12021121, Coarse-3: SRX12021109, Fine-1: SRX12021144, Fine-2: SRX12021143, Fine-3:SRX12021110). All other data have been shown in the manuscript and supplementary data.
